# Identification of candidate genes, regions and markers for pre-harvest sprouting resistance in wheat (Triticum aestivum L.)

**DOI:** 10.1186/s12870-014-0340-1

**Published:** 2014-11-29

**Authors:** Adrian L Cabral, Mark C Jordan, Curt A McCartney, Frank M You, D Gavin Humphreys, Ron MacLachlan, Curtis J Pozniak

**Affiliations:** Cereal Research Centre, Agriculture and Agri-Food Canada, Morden, MB R6M 1Y5 Canada; Crop Development Centre, University of Saskatchewan, Saskatoon, SK S7N 5A8 Canada; National Research Council of Canada, 110 Gymnasium Place, Saskatoon, SK S7N 0W9 Canada

**Keywords:** Wheat, Pre-harvest sprouting, Quantitative trait loci, Candidate genes

## Abstract

**Background:**

Pre-harvest sprouting (PHS) of wheat grain leads to a reduction in grain yield and quality. The availability of markers for marker-assisted selection (MAS) of PHS resistance will serve to enhance breeding selection and advancement of lines for cultivar development. The aim of this study was to identify candidate regions and develop molecular markers for PHS resistance in wheat. This was achieved via high density mapping of single nucleotide polymorphism (SNP) markers from an Illumina 90 K Infinium Custom Beadchip in a doubled haploid (DH) population derived from a RL4452/‘AC Domain’ cross and subsequent detection of quantitative trait loci (QTL) for PHS related traits (falling number [*FN*], germination index [*GI*] and sprouting index [*SI*]). SNP marker sequences flanking QTL were used to locate colinear regions in *Brachypodium* and rice, and identify genic markers associated with PHS resistance that can be utilized for MAS in wheat.

**Results:**

A linkage map spanning 2569.4 cM was constructed with a total of 12,201 SNP, simple sequence repeat (SSR), diversity arrays technology (DArT) and expressed sequence tag (EST) markers. QTL analyses using Multiple Interval Mapping (MIM) identified four QTL for PHS resistance traits on chromosomes 3B, 4A, 7B and 7D. Sequences of SNPs flanking these QTL were subject to a BLASTN search on the International Wheat Genome Sequencing Consortium (IWGSC) database (http://wheat-urgi.versailles.inra.fr/Seq-Repository). Best survey sequence hits were subject to a BLASTN search on Gramene (www.gramene.org) against both *Brachypodium* and rice databases, and candidate genes and regions for PHS resistance were identified. A total of 18 SNP flanking sequences on chromosomes 3B, 4A, 7B and 7D were converted to KASP markers and validated with matching genotype calls of Infinium SNP data.

**Conclusions:**

Our study identified candidate genes involved in abscissic acid (ABA) and gibberellin (GA) metabolism, and flowering time in four genomic regions of *Brachypodium* and rice respectively, in addition to 18 KASP markers for PHS resistance in wheat. These markers can be deployed in future genetic studies of PHS resistance and might also be useful in the evaluation of PHS in germplasm and breeding material.

**Electronic supplementary material:**

The online version of this article (doi:10.1186/s12870-014-0340-1) contains supplementary material, which is available to authorized users.

## Background

Preharvest sprouting is observed across all major wheat growing regions in the world. In western Canada, the average annual losses due to PHS are approximately $100 million [1]. Insufficient seed dormancy is one major factor contributing to pre-harvest sprouting losses, particularly under humid, wet weather conditions at harvest. PHS resistant/tolerant wheat cultivars and land races have been identified globally, with origins mainly in Canada, USA, Australia, China, Japan, South Africa, Kenya and New Zealand [2]. Canadian red-seeded spring wheat cultivars (AC Domain, AC Majestic, Columbus, Pasqua, Waskada, Harvest) and white spring wheat genotypes (AC Vista, Snowbird, Snowstar, Kanata, HY361) are known to carry resistance to PHS, all having derived their resistance alleles from a red-seeded breeding line RL4137 [1,3].

Of the three PHS traits, *FN* [4,5] is most commonly used to quantify PHS [6] and indirectly measures the activity of the enzyme α-amylase that breaks down starch in germinating grains. Degradation of grain-starch as the result of greater α-amylase activitys result in lower *FN* values and are an indirect indication of low levels of PHS resistance or dormancy. Two other important traits for the characterization of PHS are *GI* [7,8] and *SI* [9]. While *GI* values deduced from seed-germination tests in petri dishes are a direct measure of seed dormancy, *SI* values obtained via artificial wetting of intact wheat spikes, detect dormancy and properties of the inflorescence that affect PHS [5].

Quantitative trait loci (QTL) linked to PHS traits have been reported on all 21 hexaploid wheat chromosomes [10-13], mainly on 3A [14-17], 3B and 3D [17-19], 4A [2,20-24], 5A [25,26], 6B and 7D [27]. Of these, the PHS QTL on 4A has consistently been identified in several different mapping populations. The RL4452/‘AC Domain’ DH population has been extensively characterized for QTL detection of PHS [28], agronomic [29] and quality traits [30], in several past studies that involved a small number of molecular markers. These studies relied mainly on SSR marker data for the preparation of genetic maps and locating QTL on chromosomes. With low costs and rapid advancements in sequencing technology, thousands of molecular markers, mainly SNPs have become available in wheat. Additionally, access to genome sequence information for rice [31] and *Brachypodium* [32] will now facilitate comparative mapping for the identification of genes underlying various important quantitative traits in wheat.

Interaction among PHS QTL (QxQ, QTL epistasis), and the environment (QxE, QxQxE) have been reported from various studies [18,33-35] aimed at understanding the complex genetic structure of QTL. As chromosomal locations of PHS QTL are not uniform across populations, obtaining a consensus on the precise genomic location of important trait QTL is required for fine mapping and cloning studies. Meta-QTL or Meta-analysis [36] integrates several QTL studies of a common trait to provide a meaningful estimate of the exact location and number of QTL for that given trait. Eight PHS QTL on chromosomes 3A, 3B, 3D and 4A were identified in a Meta-QTL study [37] involving 15 different populations (five DH; nine recombinant inbred line [RIL]; one backcross [BC]).

A high level of genome-synteny exists among wheat, *Brachypodium* and rice, with wheat being more closely related to *Brachypodium* than to rice [38,39]. Conservation or collinearity of genetic markers [40,41] and greater structural similarities in the coding regions of orthologous genes [39] of wheat and *Brachypodium* have been reported. However, given differences in gene content in orthologous regions of wheat, *Brachypodium* [41] and rice [42], it might be beneficial to use both genomic sequences of *Brachypodium* and rice in comparative mapping studies for map based cloning and gene discovery in wheat.

Our study deployed SNP markers from a 90K Infinium iSelect Custom Beadchip [43], in addition to available SSR, DArT and ESTs, to generate high density genetic maps for the identification of PHS resistance QTL. Sequences corresponding to polymorphic SNPs flanking PHS QTL were analyzed against genomic sequences of *Brachypodium* and rice. The objectives of our research were a) to identify candidate genes and regions in *Brachypodium* and rice that are orthologous to PHS resistance QTL intervals in wheat, and b) to utilize sequences of SNPs flanking PHS QTL to develop KASP markers for MAS of PHS resistance.

## Results

### Linkage mapping

A total of 12,201 SNP, SSR, DArT and EST markers were mapped to all 21 wheat chromosomes. The resulting linkage map spanning 2569.4 cM is reported in Additional file 1. Of the 12,201 markers, 11,282 or 92.5% were SNPs, while the remaining 919 or 7.5% comprised SSR, DArT and EST markers. The largest number of SNP markers (6,291) were distributed across the B genome, followed by 4,125 SNPs mapped to the A genome, and 1,785 SNP markers on the D genome (Table 1).

**Table 1Table 1:** **Cumulative map-lengths of A, B and D genome chromosomes alongside corresponding genome-wise distribution of SNP markers mapped in the hexaploid DH population of RL4452/‘AC Domain’**

**Genome**	**Map length (cM)**	**Mapped markers**	**SNPs**	**SSRs, DArTs & ESTs**	**% SNPs**
A	888.4	4125	3816	309	92.5
B	940.6	6291	5871	420	93.3
D	740.4	1785	1595	190	89.4
(A + B + D)	2569.4	12201	11282	919	92.5

### QTL analysis

PHS datasets were analyzed with both MIM and simple interval mapping (SIM; data not shown) methods. As results of both methods were very similar, only those of MIM were reported in this study. The MIM [44] analysis identified four QTL with significant effects, located across chromosomes 3B, 4A, 7B and 7D. Each of these four QTL appeared in two or more environments and had peak LOD scores greater than the critical threshold LOD at 5% significance levels (α_0.05_) [45]. Coincident QTL for *GI*, *SI* and *FN* were located on chromosome 4A. Across trials, RL4452 alleles on 3B and 7B provided PHS resistance as they reduced *SI*. However, ‘AC Domain’ alleles also provided PHS resistance as they increased *FN* on 4A and 7D (with the exception of the *Glenlea 2005* trial in which they reduced *FN* on 7D) and reduced *SI* and *GI* on 4A (Table 2).

**Table 2:** **Results of Multiple Interval Mapping (MIM): four QTL for PHS traits (** ***GI, SI, FN*** **) identified on chromosomes 3B, 4A, 7B.1 and 7D.2 in a DH population of RL4452/‘AC Domain’ replicated in multi-year environments (Glenlea and Winnipeg in Manitoba; Swift Current in Saskatchewan)**

**QTL**	**Trial dataset**	**Chromosome (Linkage gp.)**	**QTL peak location (cM)**	**Additive** ^**a**^	**% PV (** ***R*** ^***2***^ **)**	**LOD**	**α0.05**
***Germination Index (GI)***							
*QGi.crc-4A*	*Glenlea2005*	4A	59.3	−0.04	27.6	12.83	3.86
*QGi.crc-4A*	*Winnipeg2004*	4A	59.5	−0.05	58.1	34.56	3.93
*QGi.crc-4A*	*Winnipeg2005*	4A	59.4	−0.02	29.6	13.93	3.86
***Sprouting Index (SI)***							
*QSi.crc-3B*	*Glenlea2005*	3B	63.6	0.43	12.7	5.39	3.96
*QSi.crc-3B*	*Winnipeg2004*	3B	70.2	0.53	16.1	6.97	3.95
*QSi.crc-4A*	*Glenlea2005*	4A	59.3	−0.57	20.5	9.12	3.96
*QSi.crc-4A*	*Winnipeg2004*	4A	56.8	−0.85	32.1	15.38	3.95
*QSi.crc-4A*	*Winnipeg2005*	4A	58.0	−0.44	12.7	5.41	3.90
*QSi.crc-4A*	*Swift Current2003*	4A	58.0	−0.49	10.5	4.41	3.94
*QSi.crc-7B*	*Swift Current2003*	7B.1	55.6	0.78	20.5	9.12	3.94
*QSi.crc-7B*	*Swift Current2004*	7B.1	56.4	0.59	11.8	4.99	3.92
***Falling Number (FN)***							
*QFn.crc-4A*	*Glenlea2005*	4A	64.2	22.49	11.2	4.71	3.83
*QFn.crc-4A*	*Winnipeg2004*	4A	56.2	45.45	25.8	11.85	3.95
*QFn.crc-7D*	*Glenlea2003*	7D.2	18.9	33.40	13.2	5.64	3.99
*QFn.crc-7D*	*Glenlea2005*	7D.2	20.2	−33.49	20.6	9.19	4.13

^a^Positive or negative additive values relate to allele effects of the AC Domain parent.

### Candidate regions and genes for PHS resistance

**Figure 1:**
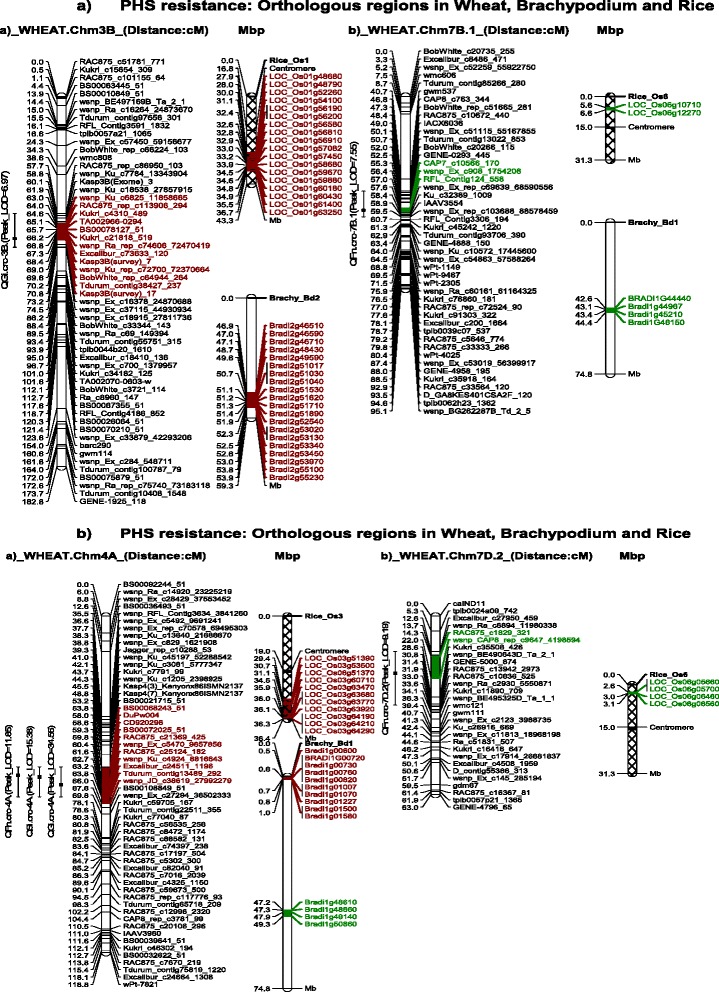
**Location of QTL and syntenic regions in** ***Brachpodium*** **and rice. a**. Location of QTL and flanking markers for PHS resistance on **a)** wheat chromosome 3B and its candidate regions on *Brachypodium Bd2* and rice *Os1*, and **b)** chromosome 7B.1 and its candidate regions on *Brachypodium Bd1* and rice *Os6.* **b**. Location of QTL and flanking markers for PHS resistance on **a)** wheat chromosome 4A and its candidate regions on *Brachypodium Bd1* and rice *Os3*, and **b)** chromosome 7D.2 and its candidate regions on *Brachypodium Bd1* and rice *Os6.*

Sequences of SNPs flanking QTL for PHS resistance on chromosomes 3B, 4A, 7B and 7D were subjected to BLASTN searches on the IWGSC and Gramene databases and returned hits to candidate regions in *Brachypodium* and rice (Table 3). Genetic and physical maps displaying orthologous regions for PHS resistance in wheat, *Brachypodium* and rice are given in Figures 1a and b. A 7.8 cM QTL interval on chromosome 3B was orthologous to a ~7.0 Mb region (46,936,013 – 53,904,697 bp) on chromosome 2 of *Brachypodium* (*Bradi2*) and to a ~8.7 Mb (27,906,608 – 36,656,340 bp) region on chromosome 1 of rice (*Os01*). The 4A QTL interval was 12.2 cM and was orthologous to a ~0.52 Mb region (481,247 – 1,030,837 bp) on chromosome 1 of *Brachypodium* (*Bradi1*) and to a ~6.9 Mb (29,401,950 – 36,320,679 bp) region on chromosome 3 of rice (*Os03*). On chromosome 7B.1, the QTL interval spanned 1.7 cM and was orthologous to a ~1.8 Mb region (42,620,688 – 44,420,413 bp) on chromosome 1 of *Brachypodium* (*Bradi1*) and to a ~1 Mb (5,588,196 – 6,603,975 bp) region on chromosome 6 of rice (*Os06*). The QTL interval on 7D.2 was 7.7 cM and was orthologous to a ~2.0 Mb region (47,249,027 – 49,335,697 bp) on chromosome 1 of *Brachypodium* (*Bradi1*), and a ~0.5 Mb region (2,558,015 – 3,079,059 bp) on chromosome 6 of rice (*Os06*).

**Table 3:** **Genetic map locations of SNP markers flanking PHS QTL on chromosomes 3B, 4A, 7B.1 and 7D.2 in a wheat DH population of a RL4452/‘AC Domain’ cross and their corresponding physical locations/candidate regions in** ***Brachypodium distachyon*** **and rice**

**SNP marker**	**Map**	**Survey sequence**	**BLASTN hits to** ***Brachypodium*** **genes**	**BLASTN hits to Rice genes**
	**(cM)**	**Contig no.**	**(genomic regions in parenthesis)**	**(genomic regions in parenthesis)**
		**Chromosome 3B**		
wsnp_Ku_c6825_11858665	63.0	10469056	Bradi2g46510 (46,936,013-46,952,333)	LOC_Os01g48680 (27,906,608-27,920,980)
wsnp_Ex_c4769_8510104	63.0	10613849	Bradi2g46590 (47,003,547-47,009,237)	LOC_Os01g48790 (27,983,688-27,990,383)
RAC875_rep_c113906_294	64.0	10557485	Bradi2g51030 (50,699,047-50,702,962)	LOC_Os01g56200 (32,367,683-32,371,816)
BobWhite_c46650_260	64.0	10441023	Bradi2g51017 (50,685,769-50,695,622)	LOC_Os01g56190 (32,350,513-32,360,765)
Kukri_c4310_489	64.6	10759762	Bradi2g51040 (50,703,620-50,708,573)	*LOC_Os02g13910* (7,558,777-7,568,835)
TA002966-0294	65.1	10635317	Bradi2g46710 (47,135,003-47,136,451)	LOC_Os01g56580 (32,615,694-32,622,894)
		10712014	Bradi2g49590 (49,632,703-49,638,684)	LOC_Os01g54100 (31,111,291-31,116,151)
BS00078127_51	65.7	10754454	Bradi2g51530 (51,119,031-51,123,083)	LOC_Os01g56810 (32,788,487-32,792,751)
Kukri_c21818_519	66.2	10455881	Bradi2g51620 (51,191,828-51,198,372)	LOC_Os01g56910 (32,869,293-32,878,216)
wsnp_Ra_rep_c74606_72470419	66.8	10523702	Bradi2g51710 (51,287,497-51,313,181	LOC_Os01g57082 (32,984,982-32,994,519)
IACX3871	66.8	10521243	Bradi2g51890 (51,441,692-51,446,044)	LOC_Os01g57450 (33,200,667-33,201,485)
Excalibur_c73633_120	67.3	10673653	Bradi2g48430 (48,731,037-48,732,308)	LOC_Os01g52260 (30,042,527-30,043,938)
wsnp_Ex_c5547_9774195	68.4	10770075	Bradi2g53020 (52,250,581-52,257,598)	LOC_Os01g59670 (34,514,117-34,520,887)
wsnp_Ex_rep_c69664_68618163	68.4	10477393	Bradi2g52540 (51,883,735-51,889,623)	LOC_Os01g58680 (33,919,393-33,924,664)
wsnp_Ku_rep_c72700_72370664	69.0	10484009	Bradi2g53340 (52,475,967-52,481,992)	LOC_Os01g60180 (34,803,492-34,804,046)
RAC875_rep_c116515_181	69.0	1068363	Bradi2g53130 (52,329,608-52,334,764)	LOC_Os01g59880 (34,629,359-34,635,205)
BobWhite_rep_c64944_264	69.6	1040995	Bradi2g53970 (52,969,054-52,973,550)	LOC_Os01g61400 (35,505,448-35,508,543)
Tdurum_contig38427_237	70.2	10658322	Bradi2g55100 (53,817,575-53,821,406)	LOC_Os01g63250 (36,656,340-36,660,768)
Tdurum_contig27495_111	70.2	10538814	Bradi2g53450 (52,567,117-52,569,109)	LOC_Os01g60430 (34,946,618-34,949,027)
Kasp3B(survey)_17	70.8	10495803	Bradi2g55230 (53,904,697-53,906,640)	*LOC_Os03g60200* (34,238,474-34,241,647)
		**Chromosome 4A**		
BS00068243_51	53.8	7023446	*Bradi2g12660* (11,006,410-11,009,518)	*LOC_Os01g28244* (15,823,709-15,829,849)
CD920298	58.6	7174272	Bradi1g00600 (481,247-482,062)	LOC_Os03g64290 (36,320,679-36,333,253)
Kukri_c12563_52	59.3	7128338	*Bradi1g51817* (50,293,482-50,308,189)	*LOC_Os05g37500* (21,943,044-21,959,786)
			Bradi1g00760 (565,638-570,467)!	LOC_Os03g63370 (35,809,964-35,814,672)!
BS00072025_51	59.3	7168762	Bradi1g00730 (555,714-559,377)	LOC_Os03g64210 (36,281,400-36,283,271)
RAC875_c21369_425	59.8	7070429	Bradi1g00820 (594,037-597,877)	LOC_Os03g64190 (36,265,672-36,271,489)
IAAV3132	59.8	7114346	Bradi1g01007 (695,876-702,209)	LOC_Os03g63920 (36,110,059-36,119,639)
wsnp_Ex_c5470_9657856	60.4	7174581	Bradi1g01070 (731,493-733,959)	LOC_Os03g51390 (29,401,950-29,403,115)
RAC875_c25124_182	61.6	7061368	Bradi1g01227 (825,624-828,017)	LOC_Os03g63680 (35,968,492-35,970,517)
wsnp_Ku_c4924_8816643	62.7	501046	*Bradi1g52230* (50,605,616-50,611,584)	*LOC_Os02g29140* (17,257,940-17,266,066)
		3540051	Bradi1G00720 (552,185-555,346)!	-
		864232	-	LOC_Os03g60710 (34,502,945-34,508,158)!
Excalibur_c24511_1196	63.2	7119833	Bradi1g49910 *(*48,564,700-48,565,690)	LOC_Os06g16640 (9,564,124-9,566,967)
		7139864	Bradi1g00820 (594,037-597,877)!	-
		5949088	-	LOC_Os03g53500 (30,679,685-30,689,230)!
Tdurum_contig13489_292	63.8	7124315	Bradi1g01500 (976,919-979,161)	LOC_Os03g63470 (35,855,445-35,860,549)
wsnp_JD_c38619_27992279	66.0	7098863	Bradi1g01580 (1,030,837-1,034,525)	LOC_Os03g63410 (35,826,263-35,830,205)
		**Chromosome 7B.1**		
CAP7_c10566_170	55.3	3116911	Bradi1G46150 (44,420,413-44,423,001)!	LOC_Os06g10710 (5,588,196-5,594,757)
BobWhite_rep_c64768_264	55.3	3032904	Bradi1G46137 (44,416,953-44,419,121)	LOC_Os06g10760 (5,619,105-5,621,750)
Tdurum_contig84962_256	55.3	3032904	Bradi1G46137 (44,416,953-44,419,121)	LOC_Os06g10760 (5,619,105-5,621,750)
BS00022498_51	55.3	3115694	Bradi1G46060 (44,341,065-44,348,362)	LOC_Os06g10880 (5,677,080-5,682,126)
wsnp_Ex_c908_1754208	56.4	3153345	Bradi1g45210 (43,434,039-43,436,397)	LOC_Os06g12270 (6,603,975-6,604,635)
Tdurum_contig68347_605	56.4	3153345	Bradi1G45210 (43,434,039-43,436,397)	LOC_Os06g12270 (6,603,975-6,604,635)
				LOC_Os06g12280 (6,605,479-6,608,454)
RFL_Contig124_558	57	3126436	Bradi1g44967 (43,073,188-43,080,744)!	-
BobWhite_c46772_564	57	3109791	Bradi1G44860 (42,951,596-42,953,323)	LOC_Os06g12990 (7,118,829-7,120,448)
			Bradi1G44850 (42,949,245-42,951,551)	-
GENE-4333_211	57	3153554	Bradi1G44790 (42,899,346-42,900,477)	-
Tdurum_contig51087_573	57	3165147	Bradi1G44440 (42,620,688-42,629,717)	LOC_Os06g13820 (7,661,691-7,670,035)
		**Chromosome 7D.2**		
RAC875_c1829_321	14.3	3849095	Bradi1g48660 (47,326,685-47,327,292)	LOC_Os06g06460 (3,040,092-3,041,121)
Kukri_c32845_116	14.3	3964075	Bradi1g50860 (49,335,697-49,339,907)	LOC_Os06g05660 (2,558,015-2,562,242)
TA002473-0717	14.3	3929478	Bradi1g49140 (47,871,489-47,874,424)	LOC_Os06g05700 (2,579,088-2,581,726)
wsnp_CAP8_rep_c9647_4198594	22.0	3945994	Bradi1g48610 (47,249,027-47,255,499)^!^	LOC_Os06g06560 (3,079,059-3,086,808)

^!^Weak hit to genomic regions in *Brachpodium* or rice that is orthologous to the QTL interval for PHS resistance in wheat.

Best hits that do not correspond to the candidate region in *Brachpodium* or rice are in italics.

*Brachypodium/*rice candidates for PHS resistance orthologous to consensus regions on wheat chromosomes 3B, 4A, 7B and 7D (Additional file 2) were identified. In the 3B region there are 895 genes in the *Brachypodium* orthologous region and 1375 in the rice region. The 4A region had 98 genes in the *Brachypodium* region and 1159 in rice, while the 7B region had 148 in *Brachypodium* and 155 in rice and the 7D region had 235 in *Brachypodium* and 88 in rice. Genes involved in ABA and GA metabolism as well as those affecting flowering time were present in the QTL regions. Among these were *Bradi2g49795/Os01g54490* (FT PEBP [phosphatidylethanolamine - binding protein] homologous to Flowering Locus T gene), *Os01g61100*, *Os01g63030* (Far-red impaired responsive [FAR1] family protein) orthologous to chromosome 3B, *Bradi1g00950/Os03g63970* (gibberellin 20 [GA20] oxidase putative expressed protein), *Os03g56630*, *Os03g62660* (Far-red impaired responsive [FAR1] family protein) orthologous to 4A, *Bradi1g46060/Os06g10880* (ABF3/ABF2 - abscissic acid responsive elements) orthologous to 7B, *Bradi1g48640*, *Bradi1g48650*, *Bradi1g48822*, *Bradi1g48816* (Far-red impaired responsive [FAR1] family protein), *Bradi1g48690*, *Bradi1g50240* (VRN1-AP2/B3 - like transcriptional factor family protein) and *Bradi1g48830*/*Os06g06320* (Vrn3/FT PEBP [phosphatidylethanolamine - binding protein] homologous to Flowering Locus T gene) orthologous to chromosome 7D.

### Development and validation of KASP primers

A total of 18 KASP markers, five each for chromosomes 3B and 7B.1, and four each for chromosomes 4A and 7D.2 (Table 4) were developed from sequences of SNPs flanking QTL for PHS resistance. Genetic map locations of individual KASP markers were identical to the respective SNP from which they were derived. Primer sets of all 18 KASP markers are listed in Additional file 3. Further, we validated the conversion of these 18 KASPs from matching genotype calls of Infinium SNP data on 183 DH progeny genotypes. Four DH progeny genotypes of the RL4452/‘AC Domain’ cross were identified to carry PHS resistance on chromosomes 3B, 4A, 7B and 7D (Additional file 4).

**Table 4:** **A list of 18 Competitive Allele-Specific PCR (KASP) markers developed for MAS of PHS from SNPs flanking PHS QTL on chromosomes 3B, 4A, 7B and 7D in a DH population of a RL4452/‘AC Domain’ cross**

**Sl.**	**KASP marker**	**Source SNP**	**Chr**	**PHS trait**
1.	Kasp3B_wsnp_Ku_rep_c72700_72370664	wsnp_Ku_rep_c72700_72370664	3B	*SI*
2.	Kasp3B_RAC875_rep_c116515_181,	RAC875_rep_c116515_181,	3B	*SI*
3.	Kasp3B_BobWhite_rep_c64944_264	BobWhite_rep_c64944_264	3B	*SI*
4.	Kasp3B_wsnp_Ex_c16378_24870688	wsnp_Ex_c16378_24870688	3B	*SI*
5.	Kasp3B_RAC875_c530_354	RAC875_c530_354	3B	*SI*
6.	Kasp4A_BS00072025_51	BS00072025_51	4A	*GI, SI, FN*
7.	Kasp4A_Kukri_c12563_52	Kukri_c12563_52	4A	*GI, SI, FN*
8.	Kasp4A_RAC875_c21369_425	RAC875_c21369_425	4A	*GI, SI, FN*
9.	Kasp4A_wsnp_Ex_c16175_24619793	wsnp_Ex_c16175_24619793	4A	*GI, SI, FN*
10	Kasp7B_wsnp_Ex_c908_1754208	wsnp_Ex_c908_1754208	7B.1	*SI*
11	Kasp7B_RFL_Contig124_558	RFL_Contig124_558	7B.1	*SI*
12	Kasp7B_RAC875_c1638_165	RAC875_c1638_165	7B.1	*SI*
13	Kasp7B_wsnp_Ex_rep_c69639_68590556	wsnp_Ex_rep_c69639_68590556	7B.1	*SI*
14	Kasp7B_Ku_c32389_1009	Ku_c32389_1009	7B.1	*SI*
15	Kasp7D_Excalibur_c22419_460	Excalibur_c22419_460	7D.2	*FN*
16	Kasp7D_RAC875_c1829_321	RAC875_c1829_321	7D.2	*FN*
17	Kasp7D_Kukri_c32845_116	Kukri_c32845_116	7D.2	*FN*
18	Kasp7D_wsnp_CAP8_rep_c9647_4198594	wsnp_CAP8_rep_c9647_4198594	7D.2	*FN*

## Discussion

The objectives of our research were to identify candidate regions for PHS resistance QTL of wheat and develop KASP markers (for MAS) from sequences of SNPs flanking such QTL. This is an important step in the process of map-based cloning of genes that underlie important quantitative traits like PHS resistance. Our objectives were achieved using 11,282 SNPs from the 90 k Infinium Custom Beadchip to develop a high density linkage map in the RL4452/‘AC Domain’ mapping population and subsequently detect QTL for PHS resistance on chromosomes 3B, 4A, 7B and 7D. Comparative mapping utilizing sequences of SNPs flanking PHS resistance QTL enabled identification of candidate genes and regions in *Brachypodium* and rice. The resulting 18 KASP markers can be deployed in future genetic studies of PHS, and in evaluation of PHS in germplasm and breeding material.

Of the 12,201 mapped markers, 11,282 or 92.5% were SNP markers, while the remaining 919 or 7.5% were SSR, DArT and EST markers. The B genome chromosomes accounted for the largest number of 6291 SNP markers, followed by the A genome with 4125 SNPs, and the D genome with 1785 SNP markers. A likely explanation for larger numbers of B genome SNP markers could be the greater genetic diversity of B genome species when compared to the A and D genome species [46,47]. A faster rate of evolution of the B genome due to greater polymorphism and duplication events, in addition to greater genetic diversity brought about by cross pollination were cited [48-50] as possible explanations for findings of a greater number of ESTs associated with more unique loci on the B genome when compared to the A and D genomes.

PHS datasets were analyzed with both MIM and SIM (data not shown) methods. Because results of both methods were very similar, only those of the MIM analyses were reported. As QTL identified using MIM were robust and supported by SIM results, it is unlikely that additional large effect QTL involved in epistatic interactions might have been detected using other QTL mapping methods that detect both main effect (M-QTL) and epistatic QTL (E-QTL). Further, a Meta-QTL study [37] reporting PHS QTL on 4A and group 3 chromosomes support significant PHS QTL identified on chromosome 3B and 4A of our study.

The most consistent of the four PHS QTL identified on chromosomes 3B, 4A, 7B and 7D were located on chromosome 4A; *GI*, *SI* and *FN* trait QTL each accounting for 58.1%, 32.1% and 25.8% of the phenotypic variation in their respective traits. The QTL for these PHS traits were coincident and maybe associated with the same gene(s). These findings might suggest that chromosome 4A is involved in regulation of PHS trait QTL in our test population. Previous reports of the association of PHS traits with chromosome 4A [2,20-24], support the importance of this QTL for PHS

In addition to a major *SI* QTL on 4A, two other QTL for *SI* were identified on chromosomes 3B and 7B.1. Both *SI* QTL on 3B and 7B.1 were detected in two of six environments. QTL that provide tolerance to late maturity α- amylase (LMA) have been mapped on 3BS and 7BL in an Australian wheat cross Cranbrook/Halberd [51]. In both studies, the SSR markers *Xwmc623, Xwmc808, Xgwm72, Xwmc612, Xgwm285, Xwmc693, Xwmc1* (3B LMA QTL interval) and *Xgwm577, Xwmc273, Xwmc276* (7B LMA QTL interval) also flanked corresponding PHS QTL intervals on chromosomes 3B.1 and 7B.1 respectively (data not shown). Further, alleles of a regulator gene *Vp-1B* on 3B have been reported to influence grain dormancy in Chinese wheat varieties [19]. In a follow up study [52], the *VP-1B* locus was validated in a white-grained Chinese landrace Wanxianbaimaizi (high seed dormancy and PHS tolerance) using SSR markers and a gene-specific primer *Vp1*. A CIM analysis identified a seed dormancy QTL *QSd.ahau-3B* on 3B flanked by *Vp1* which is linked to an SSR marker *Xwmc446* that also happens to flank the PHS QTL interval on chromosome 3B of our study. The above findings suggest that PHS and LMA QTL on chromosomes 3B and 7B are likely the same.

‘AC Domain’ alleles contributed to increasing the *FN* on 7D (linkage group 7D.2), with the exception of the *Glenlea 2005* trial, wherein a negative additive score was observed for the *FN*. While the *FN* QTL on chromosome 7D is unique to our study, a significant time to maturity (*Mat*) QTL (PV = 26%) also on 7D, and a positive contribution of the RL4452 allele, has been reported previously by [29] in the same RL4452/‘AC Domain’ population. The authors reported an SSR marker *Xgwm130* tightly linked to this QTL, which is distally located on 7DL, and is 1.1 cM from the QTL peak of our study. In the *Glenlea 2005* trial (with a negative additive score for *FN*), the average *FN* (LS Mean) score of 183 DH progeny was the lowest of the four trials (data not shown). The low *FN* score at this location might suggest greater levels of PHS of ‘AC Domain’ genotypes, probably brought on by wet weather conditions at the maturity stages or during the three weeks preceding harvest [53]. As QTL locations of both these *Mat* and *FN* traits nearly coincide and are influenced by negative and positive additive effects (with the exception of the *FN* QTL of the *Glenlea 2005* trial) of ‘AC Domain’ alleles respectively, the action of a pleiotrophic locus regulating both *FN* and *Mat* could be assumed. At Glenlea in 2005 it is possible that the lower *FN* for the Domain allele is due to adverse weather conditions at maturity or that the 7D QTL identified here might not actually be a PHS QTL, but rather a pleiotrophic effect of the *Mat* QTL on PHS.

Flanking marker intervals of a given PHS trait (*GI*, *SI* or *FN*) QTL were not always the same across trials/datasets. It is quite likely that the respective underlying genes influencing each of these traits are the same; difference in QTL interval location being mainly due to environment or experimental error from differences in class means of individual trial data sets [54]. Alternatively, the possibility of two closely linked loci controlling the same trait cannot be ruled out.

BLASTN searches with sequences of SNP markers flanking PHS QTL on chromosomes 3B, 4A, 7B and 7D revealed candidate regions in *Brachypodium* and rice genomes. The QTL interval on chromosome 3B was orthologous to regions on *Bradi2* and the long arm of *Os01*, while QTL intervals on chromosomes 4A were orthologous to regions on *Bradi1* and the short arm of *Os03*. QTL intervals on chromosome 7B.1 and 7D.2 were orthologous to regions on *Bradi1* and the short arm of *Os06* of rice. The above findings of orthology between wheat/rice chromosomes: 3B/*Os01*, 4A/*Os03* and 7B&7D/*Os06* concur with previous reports [42,55-57] of wheat/rice chromosomal region similarities revealed via comparative mapping with DNA probes and ESTs. Further, orthologies between PHS QTL intervals of 4A, 7B, 7D and genomic regions of *Bradi1*, and 3B/*Bradi2* in our study will be refined to tease out individual genes responsible for variation in PHS resistance. The availability of information on whole-genome 454 assembled gene sequences of Chinese spring [58] and gene-orthologies among the said wheat and *Brachypodium* chromosomes established using 5003 ESTs mapped to wheat deletion bins [32] will serve as useful references to complement our efforts.

Eighteen KASP markers were developed from SNP sequences flanking QTL for PHS resistance. Identical genotype calls of Infinium SNP data enabled validation of the 18 KASP markers and identified four (of 183) progeny genotypes of the RL4452/‘AC Domain’ population possessing PHS resistance on all four QTL on 3B, 4A, 7B and 7D (Additional file 4). Criteria for selection of these genotypes was based on findings of our study: ‘AC Domain’ (allele 'A') reduced *GI* and *SI* on 4A, increased *FN* on 4A and 7D, while RL4452 (allele 'B') reduced *SI* on chromosomes 3B and 7B. Further, these 18 KASP markers can be deployed in future genetic studies of PHS, and in evaluation of PHS in germplasm and breeding material.

Genes present in *Brachypodium* and rice in orthologous regions corresponding to the QTL were identified (Additional file 2). The 3B region is large and contains over 800 genes in *Brachypodium* and over 1300 in rice. More markers are needed to reduce the size of the region and the emerging reference sequence of chromosome 3B (http://wheat-urgi.versailles.inra.fr/Seq-Repository/Reference-sequence) will be a valuable resource. There are a number of ABA-inducible genes (2 Brachypodium and 3 rice) which could be a starting point to search for additional markers.

The 4A and 7B regions contain many fewer genes in *Brachypodium* and rice than the 3B region. Gibberellin 20 oxidase (GA20 – oxidase) [59] on *Bradi1/Os03* orthologous to 4A and abscissic acid responsive elements (ABF2, ABF3) [60-62] on *Bradi1/Os06* orthologous to chromosome 7B are candidates worth further study. GA20 - oxidase has previously been considered as a candidate gene underlying PHS QTL on 4A [63].

On chromosome 7D the QTL was coincident with a previously identified maturity QTL in the same population (29). Genes affecting flowering time are present in the orthologous regions in *Brachypodium* and rice. These include the Far-red impaired responsive (FAR1) related proteins [64] on chromosome *Bradi1*, as well as VRN1-AP2/B3-like transcription factors [65,66] on *Bradi1* and phosphatidylethanolamine - binding protein (PEBP) homologous to the Flowering Locus T gene [67,68] on *Bradi1/Os06*, orthologous to chromosome 7D.

Because our study utilized a large number of sequence-based SNPs not available for previous mapping studies, the resulting genetic maps and QTL flanking SNP markers are a novel and current resource for identification of underlying genes based on synteny and collinearity to model species *Brachypodium* and rice. Further, the identification of candidate genes and regions for PHS in *Brachypodium* and rice will enable a targeted focus for selection of candidate genes whose physiological/biological functions are linked to or influence variation in PHS traits under study. Such candidate gene-specific PCR markers will be developed and validated via mapping to the QTL intervals for PHS resistance in wheat.

## Conclusions

In our study we utilized SNPs from a wheat 90 K Infinium iSelect Custom Beadchip that permitted detection and assignment of significant PHS resistance QTL to specific chromosomal locations on genetic maps. Sequences of SNPs flanking PHS resistance QTL enabled identification of candidate genes and regions for PHS in *Brachypodium* and rice via comparative mapping. The 18 KASP markers resulting from this study can be suitably deployed in future genetic studies of PHS and might also be useful in the evaluation of PHS in germplasm and breeding material.

## Methods

### Plant material, experimental layout and trait phenotyping

A total of 193 DH progeny genotypes derived from a cross RL4452/‘AC Domain’ were used to develop the genetic linkage map. Of these, trait data was available on 183 DH lines for detection of QTL across the genome. Data on three PHS traits (*GI*, *SI* and *FN*) was collected from six trials: *Glenlea* (*2003*; *2005*), *Winnipeg* (*2004*; *2005*) and *Swift Current* (*2003*; *2004*), in Manitoba and Saskatchewan Canada. The phenotyping methods, experimental design and layout for each of these traits are described in [6,28].

### Molecular markers and genotyping

#### Infinium SNPs and PCR based markers

The 90 K Infinium iSelect Custom Wheat Beadchip identified 12,351 polymorphic markers that were added to existing SSR, DArT and EST markers for the RL4452/‘AC Domain’ cross. Of these, a total of 12,201 markers (11282 SNPs; 919 SSRs, DArTs and ESTs) were used in the construction of genetic maps. Further, co-segregating markers were removed from the set of 12,201 markers and QTL analysis was carried out (one marker per bin) with 1054 markers.

### Linkage mapping

Genotypic data of 193 DH progeny, screened with 12,201 markers (SSR, SNP, DArT and ESTs), were used to construct genetic maps for all 21 chromosomes. Bins of co-segregating markers were identified with MSTMap [69], and the most informative marker per bin was retained for mapping with MapDisto® [70]. Linkage groups were created using a minimum LOD score of 4 and maximum recombination fraction (*RF*) of 0.25. Recombination fractions were converted into centiMorgan (cM) map distances using the Kosambi mapping function.

### QTL analysis

Multi-year trial data collected at six environments on three PHS traits (*GI, SI, FN*) were used for QTL mapping with QGene version 3.0 software [71]. Trait data and molecular phenotypes of 183 DH progeny assessed with 1054 markers were subject to MIM and SIM (data not shown) analyses. QTL with LOD scores exceeding critical threshold values at 5% (α_0.05_), at two or more environments were deemed significant. Threshold values for trait QTL were obtained through permutation analyses involving 1000 iterations. Further, marker–trait regression (*r*^*2*^) values were interpreted as the percent phenotypic variation *(% PV*) explained due to respective QTL.

### Identification of candidate genes and regions in *Brachypodium* and rice

Sequences of SNPs flanking QTL for PHS resistance traits (*GI*, *SI*, *FN*) on chromosomes 3B, 4A, 7B and 7D were subject to a BLASTN (Basic search) on the IWGSC database (http://wheat-urgi.versailles.inra.fr/Seq-Repository). Further, best survey sequence hits were subject to a BLASTN search (Maximum E-value 10) on Gramene (www.gramene.org) against both *Brachypodium* and rice databases to obtain candidate regions for PHS resistance. QTL intervals were deduced from centiMorgan map distances between SNP markers flanking QTL peaks of a given PHS resistance trait (*GI*, *SI* or *FN*). Consensus candidate regions for PHS resistance were arrived at from best hits (of PHS QTL flanking SNP sequences) to genes and genomic regions in *Brachypodium* and rice. A few of the SNP markers returned hits to non-candidate regions/chromosomes prompting the selection of weaker hits to the consensus candidate regions. MapChart 2.2 [72] was used to construct genetic and physical maps of orthologous regions in wheat, *Brachypodium* and rice. Candidate genes in *Brachypodium* and rice corresponding to QTL intervals for PHS resistance on chromosomes 3B, 4A, 7B and 7D of wheat were obtained from the online PlantGDB database (http://www.plantgdb.org/).

### KASP markers

Sequences of SNP markers flanking QTL for PHS resistance on chromosomes 3B, 4A, 7B and 7D were converted to KASP markers. PrimerPicker Lite for KASP version 0.25 (KBioscience®) was used to generate KASP primer sets from QTL flanking SNP sequences. Protocols for the preparation and running of KASP reactions, and PCR conditions are given in the KASP manual (http://www.kbioscience.co.uk/). A FLUOstar Omega plate reader (BMG LABTECH® Offenburg Germany) with KlusterCaller™ software was used to visualize KASP marker polymorphisms.

### Availability of supporting data

All the supporting data are available as additional files.

## Additional files

Additional file 1:
**A linkage map constructed using 193 DH progeny genotypes of a RL4452/‘AC Domain’ cross evaluated with 12,201 polymorphic markers (11282 SNPs and 919 PCR markers).**
Additional file 2:
***Brachypodium*** **and rice candidates corresponding to QTL intervals for PHS resistance on chromosomes 3B, 4A, 7B and 7D in a DH population of a RL4452/‘AC Domain’ cross.**
Additional file 3:
**List of 18 Competitive Allele-Specific PCR (KASP) primers derived from sequences of SNPs flanking QTL for PHS resistance on chromosomes 3B, 4A, 7B and 7D.**
Additional file 4:
**Validation of 18 Competitive Allele-Specific PCR (KASP) markers designed from Illumina iSelect markers flanking QTL in a RL4452/‘AC Domain’ population.**
